# Summer eczema in exported Icelandic horses: influence of environmental and genetic factors

**DOI:** 10.1186/1751-0147-48-3

**Published:** 2006-05-26

**Authors:** Sigríður Björnsdóttir, Jakobína Sigvaldadóttir, Hans Broström, Birgitte Langvad, Ágúst Sigurðsson

**Affiliations:** 1 Icelandic Veterinary Services, Dept Holar, 551 Saudarkrokur, Iceland; 2 Dept Clin Sci, Sect Medicine and Surgery, Box 7018, SLU, SE-750 07 Uppsala, Sweden; 3 Valbyvej 42, 3200 Helsinge, Danmark; 4 The Icelandic Farmers Association, Hagatorgi 1, Reykjavík, Iceland

## Abstract

A cross sectional study was designed to estimate the prevalence of summer eczema (a chronic, recurrent seasonal dermatitis) in exported Icelandic horses and the influence of environmental and genetic factors on the development of the disease.

Among 330 horses, which had been exported to Germany, Denmark and Sweden, 114 (34.5%) were found to have clinical signs of summer eczema. The prevalence was highest 2 years after export and the exposure to the biting midges *Culicoides spp*., was found to be the main risk factor for developing the disease. Genetic influence on the sensitivity for the disease was not established.

It was concluded that exported Icelandic horses are predisposed for summer dermatitis and the fact that they are not introduced to the antigens of the biting midges early in live, due to it's absence in Iceland, is likely to explain the high prevalence of the disease after export.

## Introduction

Summer eczema (SE) also known as equine insect bite hypersensitivity or sweet itch, is a chronic, recurrent seasonal dermatitis of horses caused by an allergic reaction to the bite of *Culicoides ssp *midges [1-4]. The symptoms include intense pruritus, self-excoriation resulting in open wounds and secondary infections and are usually localized to the mane, tail and withers [5]. Most breeds of horses can be affected although the prevalence seems to vary considerably between countries and different regions within countries [5-10].

Due to the absence of the biting midges in Iceland [11], SE is an unknown disease in the native horse population. The disease is, however, a well-known problem in Icelandic horses that have been exported from Iceland and Icelandic horses born abroad. In an epidemiological study based on a questionnaire sent to all registered owners of Icelandic horses in Sweden, the prevalence was found to be 16.6%. It was significantly higher among horses imported from Iceland, 26.2%, compared to Icelandic horses born in Sweden, 6.7% [5]. Similar results were found for Icelandic horses in Norway, also based on a questionnaire sent to the owners [12]. The overall prevalence was 17.6%, higher for imported horses 26.9% compared to native-born horses, 8.2%.

The aim of the present study was to describe the clinical signs, estimate the prevalence and the influence of environmental and genetic factors on the development of summer eczema in exported Icelandic horses.

## Materials and methods

### Study design

A cross sectional study was designed to assess the prevalence, hereditariness and risk factors of SE in Icelandic horses that had been exported to Germany, Denmark and Sweden. Offspring of 17 sires, with the highest number of exported offspring in the previous five years and representing all the major bloodlines of the Icelandic horses (information from the Icelandic Breeding Association) were selected for the study. Owners of Icelandic horses, in the three countries, were informed about the study in local horse magazines and requested to participate with their horses. For logistical reasons, owners of big horse farms were contacted directly and their farms used as meeting points for the study.

The responding owners were visited in the years 1997–8, and all horses, fulfilling the selection criteria, were examined clinically by the same veterinarian (S.J.). Presence of skin lesions compatible with SE was recorded and the characteristics and distribution of the lesions were described. Blood samples were collected and DNA extracted from whole blood leukocytes for confirming the paternity and for further genetic studies in the future.

The owners were interviewed about the age of the horse at examination, age at export and age at first signs of SE. Information was obtained about environmental factors such as stabling, type of pasture, lee (wind) at pasture, moisture of pasture and distance from lake or running water. The owners were also asked if the area was a known inhabitation of *Culicoides spp*. or if summer eczema was a known problem for horses in the area.

### Variables

Dichotomous variables included: a) Housing (0: never in a stable or shelter, 1: shelter at pasture or in stable from the evenings to the mornings), b) Type of pasture (0: not cultivated, natural unploughed grass land, 1: cultivated ground or mixture), c) Moisture of pasture (0: dry to medium dry, 1: medium wet to swamp), d) Lee at pasture (0: mostly moderate to hard winds, 1: mostly mild to moderate winds), e) Water in the neighborhood of pasture (0: no open water within 1 km, 1: open water in the area, f) Known habitat of *Culicoides spp*. or known summer eczema area (0: no, 1: yes). Categorical variables included: gender, sire and country of examination and continuous variables included: age, age at export and years from export.

### Statistical methods

The association between the dichotomous dependent variable of the presence of SE and the potential risk factors was first screened using univariate logistic regression. Factors with P values < 0.05 were then examined further by using forward stepwise multivariate logistic regression analysis. An attempt was made to estimate the heritability of SE with REML type methods both applying a linear animal model [13] and a non-linear sire model [14].

## Results

In total 330 horses were examined for summer eczema, 170 in Germany, 82 in Denmark and 75 in Sweden. The age of the horses ranged from 1 to 25 years with the mean of 10.3 years. The distribution of gender was 58% mares, 31% geldings and 11% stallions. The age at export was known for 262 of the horses (79.4%) and ranged from few months to 16 years. The mean age at export was 3.1 years. Time since export was known for 312 of the horses (94.5%) ranging from few months to 22 years with the mean of 4.7 years.

Clinical signs of SE were found in 114 horses or 34.5%. Information about the time of onset of the disease after export was available for 49 of the affected horses and ranged from 1 to 8 years. In most cases the onset of SE was first detected 2 years after export (mean 2.4 years) (Table 1). For horses exported for more than two years prior to the study (n = 213), SE was diagnosed in 49.5%.

**Table 1:** Number of years from export until the first clinical signs of SE were detected in 49 Icelandic horses.

**Years from export**	**0**	**1**	**2**	**3**	**4**	**5**	**6**	**7**	**8**
**New cases of SE**	7	8	16	8	4	3	1	1	1
**Prevalence of SE %**	14	16	33	16	8	6	2	2	2

The characteristics and distribution of the skin lesions was described for 43 of the horses. The most common signs were itching (100%), thickening of the skin (100%), alopecia (97%), excoriation (91%), scale (89%) and wounds (71%). Secondary infections were not described. The symptoms were recorded at the mane (93%), tail (72%), abdomen (30%), head (21%), side (9%) and chest (5%).

### Risk factors

The univariate logistic regression analysis revealed a significant association between the presence of SE and the variables: age, years from export, known habitat of *Culicoides spp*., cultivation of pasture, moisture of pasture, lee at pasture and open water in the neighborhood of pasture (Table 2). The five last variables are all supposed to reflect the same risk factor: the exposure to the biting insect *Culicoides spp*. The final multivariate model (Table 3) includes the variables: years from export, moisture of pasture and lee at pasture.

**Table 2:** 

**Risk factors/variables**	**Information rate**	**N**	**n**	**(%)**	**p**
*Age*	84,2				0,000
*Age of export*	79,4				0,200
*Years from export*	94,5				0,002
*Gender*	95,8				0,671
Geldings		99	34	(34)	
Mares		183	62	(34)	
Stallions		34	10	(29)	
*Sire*	100				0,696
0		123	43	(35)	
1		10	2	(20)	
2		19	5	(26)	
3		19	9	(50)	
4		10	3	(30)	
5		23	8	(35)	
6		10	8	(80)	
7		28	10	(36)	
8		8	3	(38)	
9		9	1	(11)	
10		4	1	(25)	
11		8	3	(38)	
12		14	6	(43)	
13		9	0	(0)	
14		13	2	(15)	
15		4	1	(25)	
16		5	2	(40)	
17		15	7	(47)	
*Country of examination*	100				0,182
Sweden		75	25	(33)	
Danmark		84	24	(29)	
Germany		171	65	(38)	
*Culicoides inhabition*	82,1				0,000
No		147	34	(23)	
Yes		124	57	(46)	
*Housing*	82,7				0,161
No		132	39	(30)	
Yes		141	53	(38)	
*Cultivation of pasture*	83.0				0,025
No		110	46	(42)	
Yes		164	47	(29)	
*Moisture at pasture*	82,1				0,000
No		156	37	(24)	
Yes		115	52	(45)	
*Lee at pasture*	82,1				0,000
No		94	17	(18)	
Yes		177	72	(41)	
*Open water at pasture*	82,4				0,01
No		143	38	(27)	
Yes		129	52	(40)	

**Table 3:** Final model based on multivariate logistic regression of possible risk factors of SE for for 275 Icelandic horses.

**Risk factors**	**OR**	**CI (95%)**	**p value**
Years from export*	1,097	1,016 – 1,183	0,017
**Moisture at pasture**	1,860	1,023 – 3,366	0,040
**Lee at pasture**	2,278	1,155 – 4,491	0,017

* Odds-Ratio (OR) and 95% confidence intervals (CI) for each year

The estimated heritability was not significantly different from zero.

## Discussion

At the time of the examination a central database did not exist for Icelandic horses in Europe prohibiting selection of a random material from the population. The central database [15] introduced in 2000 has improved the availability of information about the population. The wide distribution of exported Icelandic horses in Europe, where they are kept in different environments and where the management is variable, make a complete epidemiological study difficult to perform. The owners are, however, well aware about the disease and were willing to participate with their horses in the survey. Emphasis was made on visiting large horse farms where all horses fulfilling the criteria were examined. On these farms, horses were kept from many owners who were not all able to be there personally at the time of the examination. This is the main reason for some missing data of the questionnaire.

The prevalence was higher in the present study (34.5%) compared to results earlier reported from Sweden and Norway [5,12]. The main reason is that only horses exported from Iceland were included in our study. Clinical examination of the horses might also have contributed to higher prevalence compared with studies based on questionnaires sent to the owners as mild symptoms of SE, probably overseen by the owners, were revealed. There is also a risk that the current material might be biased as owners of horses with SE or living on areas where SE is a problem were probably more willing to participate in the study.

Although comparable figures are not available for other breeds, the high prevalence found in this study indicates that Icelandic horses that have been exported to the continent of Europe are predisposed for SE. The export of horses is followed by great environmental changes including different weather conditions and introduction to new microbes and insects. Together with stress, due to the transport, these changes seem to influence the immune system negatively.

Number of years after export influenced the prevalence of the disease. The prevalence in this study was highest 2 years after export, which gives one year shorter sensitization period than earlier reported [12]. The different methods used in these studies can explain this as earlier stages of the disease might have been detected by clinical examination compared to questionnaire sent to the owners. Age at export did on the other hand not influence the prevalence of SE and neither did country of import or gender.

The exposure to the biting insect *Culicoides spp*., seems to be the main risk factor for developing SE after export. For horses that were exported for more than two years ago and living in a known habitat of the responsible insect, the prevalence of SE was as high as 54%. The variables, moisture of pasture and lee at pasture seem to be basic factors for the inhabitation of the insect at pasture. The fact that Icelandic horses are not introduced to their antigens in early live is likely to explain the high prevalence in exported horses. In laboratory animals it has been shown that allergic IgE responses to certain antigens can be specifically suppressed by passive transfer of maternal immunity via colostrum from immunised mothers [16,17].

There were no evidences of sire effect on the prevalence of the disease in this material and the estimated heritability was not different from zero. The genetic influence on the disease has, however, not been ruled out. Due to the great influence of environmental factors, the material must be regarded as far too small for detecting the genetic effects [18].

The description of the clinical signs was in agreement with earlier reports [5,19].

According to current knowledge, the prevention of the disease must be based on avoidance of the biting midges, keeping the horses on windy, dry pasture, stabling the horses when the midges are most active or using eczema blankets.
